# Effects of sleep on multimodal cognitive functioning in college students

**DOI:** 10.3389/fpsyt.2025.1637699

**Published:** 2025-12-02

**Authors:** Yubing Xu, Linglong Qin, Shiman Wen, Xuanyu Zhang, Yanling Zhou

**Affiliations:** 1 School of Mental Health, Guangzhou Medical University, Guangzhou, China; 2 The Affiliated Brain Hospital, Guangzhou Medical University, Guangzhou, China; 3 Guangdong Engineering Technology Research Center for Translational Medicine of Mental Disorders, Guangzhou, China

**Keywords:** college students, sleep, cognitive function, insomnia, generalized linear model

## Abstract

**Objective:**

While sleep disturbances are known to be associated with cognitive decline, the impact of sleep duration and sleep disturbances on cognitive functions remains poorly characterized in college students. This study aimed to quantify the prevalence of insomnia and poor sleep quality in a large sample of college students, and to elucidate the link between sleep disturbances and declines in different cognitive functions.

**Methods:**

The sample consisted of 1021 college students who completed a web-based survey containing the Pittsburgh Sleep Quality Index (PSQI), Insomnia Severity Index (ISI), and Multimodal Cognitive Ability Self-Assessment Scale (MASQ). Data were analyzed using one-way analysis of variance (ANOVA) and generalized linear models (GLM).

**Results:**

A total of 54.1% of college students reported insomnia, and 58.9% of the sample had poor sleep quality. Regarding sleep subgroups, shorter sleep duration was significantly associated with more severe sleep disturbances, including poor sleep quality (p<0.001), insomnia (p<0.001), difficulty falling asleep (p=0.001), daytime dysfunction (p<0.001), and Delayed Sleep-Wake Phase Disorder (DSWPD) (p<0.001). These sleep disturbances were associated with poorer cognitive performance. Furthermore, sleep disturbances, including difficulty falling asleep, difficulty staying asleep, waking up too early, daytime dysfunction, and DSWPD, were negatively associated with different cognitive functions: language, visual-perceptual ability, verbal memory, visual-spatial memory, and attention/concentration.

**Conclusion:**

The results of this study suggest that insomnia and poor sleep quality are common in college students, and short sleep duration exacerbates sleep disturbances, a combination that can lead to declines in cognitive functions.

## 1 Introduction

Sleep plays a crucial role in optimizing cognitive functioning, contributing to physical recovery, memory consolidation, learning, and emotional regulation (1). Sleep disturbances are very common among college students nowadays. These disturbances are specifically manifested as staying up late, daytime dysfunction, irregular sleep time, which can affect academic performance and mood (2). As the most common circadian rhythm disorder among adolescents, Delayed Sleep-Wake Phase Disorder (DSWPD) carries a prevalence of 7%–16% and imposes a significant detrimental impact on quality of life (3). Meanwhile, DSWPD is highly comorbid with mental health issues such as depression, anxiety, and self-injury, highlighting its significant public health relevance (4). Despite its typical onset in adolescence, there is limited understanding of its persistence in early adulthood.

Numerous life activities and physiological functions are influenced by sleep. Sleep disturbances can lead to various physiological dysfunctions, including impairments in cognitive functioning and abnormalities in emotional health. According to a study from USA, insufficient sleep impairs cognitive functioning by affecting dendritic spines, and this damage is irreversible (5). A study based on UK Biobank demonstrated that sleep-related symptoms are directly associated with slower reaction times and lower scores on cognitive functions such as fluid intelligence, working memory, and executive function (6). A Chinese study focusing on adolescents revealed that unhealthy sleep patterns can prospectively predict an increased future risk of depression (7). Insomnia and sleep disturbances are key bridge symptoms connecting different psychological issues like depression and anxiety.

The neural mechanisms by which sleep influences cognitive function are complex. Research suggests this is an active process involving multiple synergistic systems. Central to this process is the astrocyte circadian clock, which maintains brain homeostasis by facilitating toxin clearance, synaptic resetting, neurotransmitter balance, and inflammation suppression (8, 9). Simultaneously, the suprachiasmatic nucleus (SCN) stimulates melatonin production from the pineal gland. This melatonin, in turn, acts on the SCN to stabilize circadian rhythms. Through these actions, the system not only improves sleep quality but also directly contributes to neuroprotection by clearing brain toxins, consolidating memory, and countering inflammation and oxidative damage (10–13). From a macroscopic circulation perspective, high-quality sleep (indicated by lower PSQI scores) enhances cerebrospinal fluid clearance along the olfactory nerve pathway, particularly via the inferior turbinate. This enhanced clearance efficiently removes metabolic waste and inflammatory factors from the brain (14, 15). Together, these coordinated mechanisms effectively delay cognitive decline, support cognitive functions such as learning, memory, and attention, and preserve long-term brain health.

It is crucial to investigate the sleep status of college students and to further explore its relationship with cognitive functioning. This study aims to assess the current sleep conditions among this population. By analyzing the relationship between sleep and cognitive functioning, we seek to inform the development of effective interventions against sleep disturbances and cognitive decline.

## 2 Methods

### 2.1 Study designs and participants

The sample size was calculated using the formula:


$$\text{n}=\frac{{\text{Z}}^{2}\times \text{p }(1-\text{p})}{{\text{e}}^{2}}$$

p represents the poor sleep quality detection rate among college students, which is 0.69 (16), and e denotes the allowable error, which determines the maximum acceptable difference between the sample rate and the over-all rate. The allowable error was set to 0.05. Moreover, α represents the desired significance level, which was set to 0.05 for a 95% confidence interval, with the corresponding value of Z being 1.96. The calculated sample size was 168.

**Figure 1:**
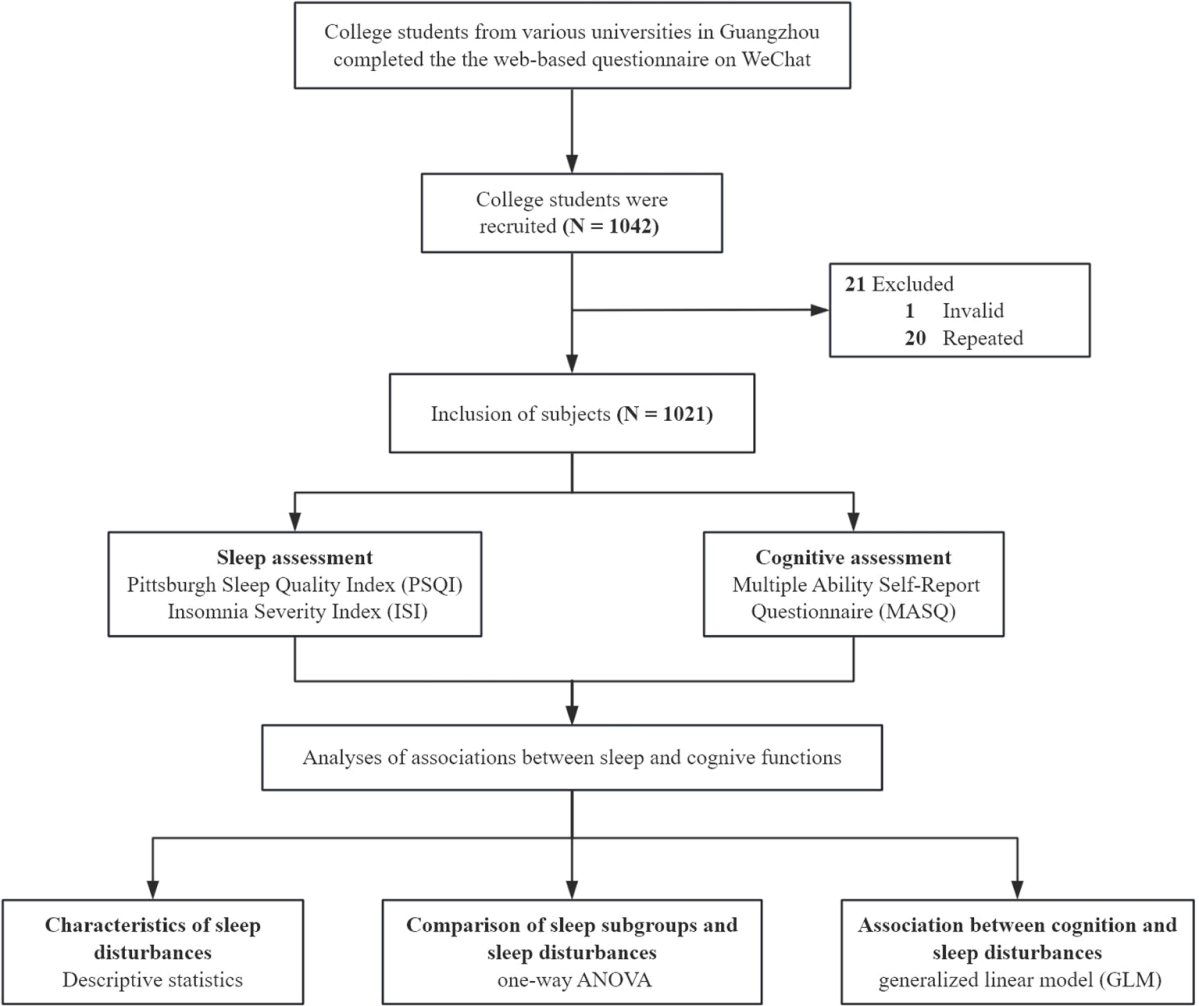
Study flowchart.

Through open recruitment, a web-based questionnaire was administered to college students from multiple universities in Guangzhou. The data collection period spanned from April 2024 to April 2025. Participation was voluntary, and all participants signed an informed consent form.The confidentiality of participants’ information and responses was strictly maintained. From a total of 1,142 submitted questionnaires, 1,021 were deemed valid and included in the study following the removal of invalid responses. The questionnaires included the Pittsburgh Sleep Quality Index (PSQI), Insomnia Severity Index (ISI), and Multiple Ability Self-Report Questionnaire (MASQ). The flowchart of the study is shown in **Figure 1**.

### 2.2 Tools

#### 2.2.1 PSQI

PSQI is a one-month questionnaire assessing sleep quality (17). It consists of 19 items that evaluate various aspects of sleep during the past month. These items generate seven component scores, with higher scores indicating more severe sleep disturbances. If the total score is higher than 5, the quality of sleep is poor.

Based on the National Sleep Foundation (NSF) recommendations for sleep duration (18), sleep subgroups were classified using PSQI entry 4: (i) short: PSQI4 < 7h for deficient (not recommended); (ii) adequate: 7h ≤ PSQI4 < 9h for appropriate (recommended); and (iii) long: PSQI4 ≥ 9h for suboptimal (may be too long).

Sleep disturbances included difficulty in falling asleep, use of sleeping medication, and daytime dysfunction. These were determined by scores on different components of the PSQI. Difficulty falling asleep was defined as a score of ≥2 on the PSQI component B, use of sleep medication as a score of ≥2 on the component F, and daytime dysfunction as a score of ≥2 on the component G. DSWPD was identified according to PSQI entry 1 and component G. Specifically, at two and beyond on PSQI entry 1, combined with a score of ≥2 on component G, was used to define DSWPD (19). For each criterion, a score of 1 indicated the presence of the disturbances, while 0 indicated its absence.

#### 2.2.2 ISI

ISI is a self-rating scale used to assess an individual’s insomnia symptoms over the past two weeks, with the higher the scores indicating the more severe the disturbances (20). Based on the total ISI score, insomnia severity was categorized as follows: no clinically significant insomnia (< 8), subthreshold insomnia (8~14), clinical insomnia of moderate severity (15~21), and clinical insomnia of severe severity (22~28). In the sleep disturbances, difficulty staying asleep was defined as a score of ≥2 on ISI entry 2 (21), while disturbances with waking up too early were defined as a score of ≥3 on ISI entry 4 (22). If the disturbance exists, it will be scored as 1, if not, it will be scored as 0.

#### 2.2.3 MASQ

MASQ is a self-report measure containing items from five cognitive domains: language, visual-perceptual ability, verbal memory, visual-spatial memory, attention/concentration (23). It can be used as a measure of cognitive function, with the higher scores indicating the more severe the cognitive impairment.

### 2.3 Statistical analysis

Descriptive statistics were used to characterize the data. The scores for sleep disturbances were expressed as mean and standard deviation, where a score closer to 1 indicates a more severe disturbance. One-way ANOVA was used to compare sleep disturbances among the different sleep subgroups and to make comparisons across genders, followed by a *post hoc* Tukey HSD test to determine significant differences. Bonferroni corrections were used to handle multiple comparisons. Generalized linear models (GLM) were used to analyze the relationship between sleep duration, sleep disturbances, and cognitive function. Covariates such as gender, age, major, and grade were included to control for potential confounding. Robust standard errors were incorporated to ensure valid inference under heteroscedasticity. Multicollinearity among independent variables was assessed with variance inflation factor (VIF), with no evidence of multicollinearity was found. The assumptions of linear regression were verified through tests of skewness and kurtosis and examination of residual plots. Cook’s distance was used to identify observations potentially affecting the regression model to ensure that the model was robust and reliable. The significance level for this study was set at p<0.05. Analyses were performed using IBM SPSS 25 and the R 4.5.0 software.

## 3 Results

### 3.1 Demographics and sample characteristics

The STROBE checklist for the study is displayed in **Supplementary Table 1**. Insomnia was reported by 54.1%, and poor sleep quality was reported by 58.9%. The most prevalent sleep disturbances were difficulty falling asleep (36.0%) and daytime dysfunction (70.3%). The prevalence of difficulty staying asleep was 17.0%; waking up too early was 11.9%; DSWPD was 13.2%. The least prevalent sleep disturbance was use of sleeping medication (4.2%). The results of descriptive statistics on sleep subgroups and the prevalence of sleep disturbances among college students are shown in **Table 1**.

**Table 1:** Demographic and sleep characteristics of the sample (N = 1021).

Variables		Mean ± SD/N(%)
Demographic	Age	20.29 ± 1.47
	Gender	
	Female	736 (72.1%)
	Male	285 (27.9%)
	Major	
	Medical majors	551 (54.0%)
	Non-medical majors	470 (46.0%)
	Grade	
	Freshmen	210 (20.6%)
	Sophomore	338 (33.1%)
	Junior	300 (29.4%)
	Senior	120 (11.7%)
	Final	31 (3.0%)
	Postgraduate	22 (2.2%)
Sleep characteristics	Sleep quality according to PSQI	6.44 ± 2.94
	Good sleep quality	420 (41.1%)
	Poor sleep quality	601(58.9%)
	Insomnia severity according to ISI	8.67 ± 4.65
	No clinically significant insomnia	469 (45.9%)
	Subthreshold insomnia	431(42.2%)
	Clinical insomnia of moderate severity	117 (11.5%)
	Clinical insomnia of severe severity	4 (0.4%)
	sleep disturbances	
	Difficulty falling asleep	368 (36.0%)
	Use of sleeping medication	43 (4.2%)
	Daytime dysfunction	710 (69.5%)
	Difficulty staying asleep	174 (17.0%)
	Waking up too early	121 (11.9%)
	DSWPD	135 (13.2%)

DSWPD, Delayed Sleep-Wake phase disorder; PSQI, Pittsburgh Sleep Quality Index; ISI, Insomnia Severity Index; MASQ, Multimodal Cognitive Ability Self-Assessment Scale.

### 3.2 Comparison of sleep subgroups and sleep disturbances

Significant correlations were observed between the sleep subgroups and several sleep disturbances, including sleep quality, insomnia severity, difficulty falling asleep, daytime dysfunction, and DSWPD, showing minor differences between males and females.

Among all participants, daytime dysfunction demonstrated a graded relationship with sleep duration: the short sleep subgroup had the worst sleep disturbances, the adequate sleep subgroup was suboptimal, and the long sleep subgroup was the least affected. Scores for sleep quality, insomnia severity, and difficulty falling asleep were significantly higher in the short sleep subgroup compared to both the adequate and long sleep subgroups, and scores for waking up too early were significantly higher in the short sleep subgroup than in the adequate sleep subgroup. Furthermore, the scores for DSWPD was significantly higher in both the short and long sleep subgroups than in the adequate sleep subgroup (**Table 2**).

**Table 2:** Results of intergroup comparisons of sleep disturbances among sleep subgroups.

Sleep characteristics	Sleep subgroups			*Post-hoc* ^a^	ANOVA p-value
	Short (N = 359)	Adequate (N = 576)	Long (N = 86)		
Sleep quality	0.82 ± 0.39	0.48 ± 0.50	0.38 ± 0.49	S>A, L	<0.001
Insomnia Severity	0.66 ± 0.48	0.49 ± 0.50	0.40 ± 0.49	S>A, L	<0.001
Difficulty falling asleep	0.43 ± 0.50	0.32 ± 0.47	0.30 ± 0.46	S>A, L	0.001
Use of sleeping medication	0.05 ± 0.22	0.04 ± 0.19	0.05 ± 0.21	/	0.586
Daytime dysfunction	0.84 ± 0.37	0.64 ± 0.48	0.47 ± 0.50	S>A>L	<0.001
Difficulty staying asleep	0.20 ± 0.40	0.16 ± 0.37	0.13 ± 0.34	/	0.177
Waking up too early	0.15 ± 0.36	0.10 ± 0.31	0.09 ± 0.29	S>A	0.101
DSWPD	0.19 ± 0.39	0.09 ± 0.28	0.20 ± 0.40	S, L>A	<0.001

DSWPD, Delayed Sleep-Wake phase disorder; S, short sleep subgroup; A, adequate sleep subgroup; L, long sleep subgroup.

a Tukey HSD *post-hoc* tests, significance level set to 0.05.

In females, daytime dysfunction also demonstrated a graded relationship with sleep duration. Scores for sleep quality and insomnia severity were significantly higher in the short sleep subgroup compared to both the adequate and long sleep subgroups, and scores for difficulty falling asleep were significantly higher in the short sleep subgroup than in the adequate sleep subgroup. Similarly, the scores for DSWPD was significantly higher in both the short and long sleep subgroups than in the adequate sleep subgroup. A similar pattern was observed in males. In contrast, there were no significant differences in DSWPD scores among the different sleep subgroups (**Supplementary Table 2**).

### 3.3 Association between cognitive functions and sleep disturbances

**Figure 2:**
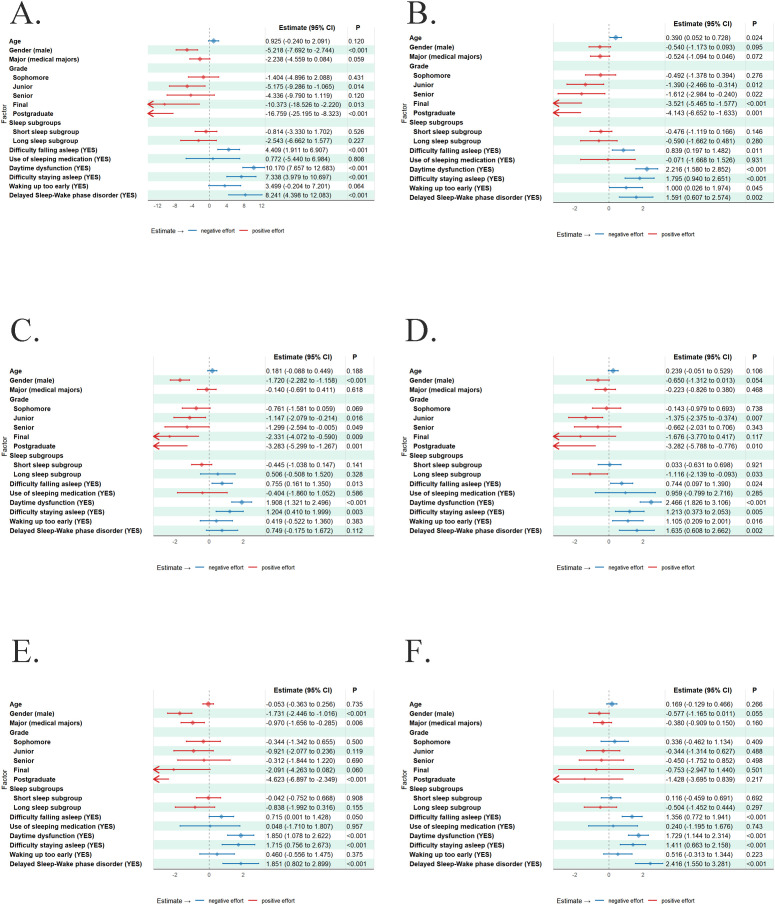
Association between sleep disturbances and: **(A)** overall cognitive function; **(B)** language; **(C)** visual-perceptual ability; **(D)** verbal memory; **(E)** visual-spatial memory; **(F)** attention/concentration. The horizontal lines represent the 95% confidence intervals(CI). Red horizontal lines indicate a positive effect relative to the reference group, meaning better performance in cognitive function; blue horizontal lines indicate a negative effect, meaning worse performance. Diamonds represent the effect sizes, while arrows indicate the direction of 95% CI that extend beyond the axis limits. Effects are considered statistically significant (p-value < 0.05) if their 95% CI do not include zero.

To ensure robust inference in the presence of heteroskedasticity, we employed generalized linear models with robust standard errors, along with several diagnostic tests, to examine the relationships between sleep subgroups and disturbances and cognitive function. Forest plots were used to visualize the relationships between sleep disturbances and cognitive functions for which significant correlations were identified (**Figure 2**).

Higher scores on difficulty falling asleep, daytime dysfunction, difficulty staying asleep, and DSWPD were significantly negatively correlated with overall cognitive function, which means participants who had these disturbances performed worse in overall cognitive function (**Figure 2A**). Among different kinds of cognitive functions, higher scores on difficulty falling asleep, daytime dysfunction, difficulty staying asleep, waking up too early, and DSWPD were significantly negatively correlated with cognitive function in language (**Figure 2B**). Higher scores on difficulty falling asleep, daytime dysfunction, and difficulty staying asleep were significantly negatively correlated with cognitive function in visual-perceptual ability (**Figure 2C**). Longer sleep duration (≥9h) were significantly positively correlated with cognitive function comparing with short sleep subgroup, and higher scores on difficulty falling asleep, daytime dysfunction, difficulty staying asleep, waking up too early, and DSWPD were significantly negatively correlated with cognitive function in verbal memory (**Figure 2D**). Higher scores on difficulty falling asleep, daytime dysfunction, difficulty staying asleep, and DSWPD were significantly negatively correlated with cognitive function in visual-spatial memory (**Figure 2E**). Higher scores on difficulty falling asleep, daytime dysfunction, difficulty staying asleep, and DSWPD were significantly negatively correlated with cognitive function in attention/concentration (**Figure 2F**).

## 4 Discussion

### 4.1 Sleep of college students in general

This study reveals a concerning prevalence of sleep disorders among college students, with over half reporting insomnia (54.1%) and a majority experiencing poor sleep quality (58.9%). The problem is further compounded by a pervasive pattern of insufficient sleep, with average durations consistently falling below the 7-hour recommendation. This finding aligns with a study highlighting a crisis in college student sleep health (16).

Among the sleep disturbances, daytime dysfunction emerged as exceptionally widespread (70.3%), suggesting that sleep issues profoundly impact students’ daytime activities, while difficulty falling asleep (36.0%) points to significant challenges with sleep initiation. The profile of sleep disturbances identified in this cohort, which was characterized by high rates of poor sleep quality and excessive daytime sleepiness, is consistent with patterns observed in other college student populations (24).

Collectively, these results underscore that sleep disturbances are not isolated incidents but a common and debilitating challenge within the college demographic, demanding targeted intervention.

### 4.2 Effort of sleep duration in sleep disturbances

In the ANOVA, the short sleep subgroup performed worse than the adequate and long sleep subgroups across most sleep disturbances. This indicates that a sleep duration of ≥ 7 hours is critically associated with good sleep quality in college students, while ≥ 9 hours or more has a limited impact. Furthermore, the prevalence of DSWPD was significantly lower in the adequate than in the short and long sleep subgroups among females. When a person’s social time is out of sync with their circadian timing, they may be considered to have a circadian rhythm disorder or social jetlag. Social jetlag refers to the discrepancy in sleep habits between work and free days, and the conflict between social and biological time (25). A previous study has shown that in younger populations, sleep differences between weekdays and weekends are becoming more prominent during the school year and may contribute significantly to daily sleep differences, especially during the school year (26). Moreover, female students consistently demonstrated better habits of attending school on time, which may contribute to a greater likelihood of social jetlag. We presume that the college students with DSWPD are more prone to extremes in sleep duration especially in females, and therefore less likely to be classified in the adequate sleep subgroup. Social jetlag is associated with a range of cognitive impairments, including impulsivity, inattention, and diminished attentional/inhibitory performance (27, 28).

Sleep disturbances are associated with numerous factors categorized into biological, psychological, and social factors. Biological factors include circadian rhythm disruption, decreased melatonin secretion, increased cortisol secretion, and poor lifestyle habits. In patients with DSWPD, DSWPD coexists with reduced sleep duration, difficulty falling asleep, and decreased sleep efficiency, indicating its broad disruption of both sleep length and quality (29, 30). Nocturnal use of electronic devices suppresses melatonin secretion (31), leading to difficulty falling asleep and reduced sleep duration (32). In terms of psychological factors, various emotions such as depression, anxiety, stress and learning burnout have demonstrated relationships with sleep. Core symptoms of depression like “fatigue” and “low energy” correspond to daytime dysfunction directly (33). “Sleep disturbances” and “insomnia” are key symptoms connecting the networks of depression, anxiety, and sleep quality (34). Learning burnout is associated with daytime dysfunction and sleepiness (35). Stress is linked to difficulty falling asleep and exacerbates sleep disturbances (36). Regarding social factors, a discordant family environment and poor family relationships can affect sleep negatively, exacerbating sleep disturbances and reducing sleep quality (37). Community stress, neighborhood disorder, and a lack of community safety are linked to various sleep issues, including poor sleep quality, difficulty staying asleep, and daytime dysfunction (37).

In summary, sleep disturbances among college students are associated with complex factors including biological, psychological and social factors. Biologically, we identified a linkage between insufficient sleep duration and sleep disturbances, which is affected by circadian misalignment and electronic device use. Psychologically, these sleep issues are deeply intertwined with emotional and academic distress, such as depression and learning burnout. Socially, factors ranging from family discord to community environment directly impact sleep quality and daytime function. This pervasive interplay between sleep, cognition, and multifaceted risk factors underscores the critical need for integrated intervention strategies that address not only sleep habits but also the underlying psychological well-being and social context of college students.

### 4.3 Sleep disturbances and cognitive decline

The present study found that sleep disturbances, such as difficulty falling asleep, daytime dysfunction, difficulty staying asleep and DSWPD, had significant positive correlations with declining cognitive functions. Among them, difficulty falling asleep, daytime dysfunction, and difficulty staying asleep were important factors in cognitive decline, with significant positive correlations in all six types of cognitive decline, including overall cognitive function, language, visual-perceptual ability, verbal memory, visual-spatial memory, and attention/concentration. In older adults, there was a positive correlation between cognitive impairment and increased daytime dysfunction (38). In addition, difficulty falling asleep is a significant association of declines in language, visual-perceptual ability, and attention/concentration, a finding that is consistent with other results linking patients with insomnia with objectively determined findings to impaired attentional shifts and working memory (39). Among college students, visuospatial learning and memory performance declines as average sleep quality declines (40). DSWPD is a significant related factor of decline in language, verbal memory, visual-spatial memory and attention/concentration decline. DSWPD due to the mismatch between social and physiological time maladjustments would in turn lead to chronic sleep debt from late bedtimes and early risings, which in turn would alter cognitive performance and have consequences for attention (41). Although there is no significant correlation between waking up too early and overall cognitive function, there is a significant correlation with language and verbal memory. Altered sleep patterns may affect language function by influencing the connectivity of brain-related functions, with effects on language-related brain networks varying by gender and age (42).

In our study, there was almost no significant association between sleep duration and cognitive function. However, there are many possibilities for factors affecting the correlations. In young adults aged 18-35, with the sleep duration reduced, it didn’t lead to significant differences in cognitive function among the participant, which is a result consistent with our findings. But during cognitive task, a metric of rapid reorganization within brain modules as measured by flexibility, significantly increased as sleep duration decreased, seems that the brain appears to compensate for the cognitive challenges posed by declining sleep durations by reorganizing dynamic functional connectivity, thereby preserving behavioral performance (43). Young adults who have experiencing sleep deprivation show enhanced connectivity between the anterior cingulate cortex and the insula during vigilance tasks (44). These findings suggest the presence of compensatory neural activity in the brain.

As age increases, among middle-aged and older adults aged over 40, a correlation between sleep and cognition directly. Sleep acts as a bridge between cognition and depression, exhibiting an inverted U-shaped curve, where both excessively short and long sleep durations are associated with cognitive impairment (45). Among older adults, the relationship between sleep and cognition becomes more significant, exhibiting a bidirectional association. Sleep is linked to the risk of impaired cognitive function, while sleep disturbances can serve as early manifestations or risk factors for cognitive disorders such as dementia. Similarly, an inverted U-shaped relationship exists between sleep and cognition: sleep durations exceeding 8 hours are negatively correlated with cognitive function, whereas 6–8 hours of sleep show a positive correlation (46). Insufficient sleep impairs the clearance of metabolic waste products in the brain, such as β-amyloid (47). Prolonged sleep duration (≥8 hours) is associated with an increased risk of dementia and represents an independent risk factor for the condition. Poor sleep quality adversely affects cognitive flexibility, attention, processing speed, and other executive domains (48). Notably, cognitive impairment may precede prolonged sleep duration, with increased sleep time observed as early as 6–10 years prior to a dementia diagnosis (46). Therefore, sleep may function both as a cause of cognitive decline and as an early indicator of cognitive impairment. Among patients with Alzheimer’s disease (AD), in the mild cognitive impairment (MCI) stage, sleep disturbances worsen, manifesting as reduced slow-wave sleep during NREM and shorter total sleep time (49). Children with neurodevelopmental disorders (NDD) commonly experience more frequent and severe sleep problems, which interact bidirectionally with the disorders themselves and significantly impact the children’s cognition and behavior (50).

Based on these previous studies, we hypothesize that the neurobiological mechanisms of sleep may influence the relationship between sleep and cognition at different life stages. Cortical excitability, which is closely tied to sleep-wake regulation, decreases with prolonged wakefulness (51). During sleep deprivation, cortical excitability is higher and dynamically diminished in older adult. Conversely, cortical cortical excitability is lower and more flexible to change in younger adults. In older adults, this pattern potentially suggests a decline in brain adaptation that adversely affects cognitive flexibility. In young adults, however, a positive anisotropic response to acute sleep-wake disruption appears to serve as a protective mechanism by enhancing the counteraction of homeostatic sleep pressure, ultimately manifesting as cognitive health (52). Although sleep duration shows no significant association with cognitive behavioral performance in young adults, underlying neural compensatory mechanisms are at play. This adaptive mechanism is particularly prominent in younger brains and may decline with age. In addition, biological aging due to persistent oxidative stress and inflammation, among others, is also associated with cognitive decline with age (53).

While sleep quality and insomnia are not directly correlated with cognitive functions in college students, sleep disturbances still relate to cognitive decline. Thus, a close relationship between sleep and cognition persists. Therefore, sleep health plays an important role in cognitive functioning, and early intervention is potentially valuable in preventing cognitive decline in later life. Future research could further explore these relationships and how different kinds of cognitive functions can be maintained and enhanced by improving sleep habits and treating sleep disorders.

### 4.4 Limitation

There are some limitations of this study. First, our study design used a cross-sectional study, which did not allow us to make claims of causal relationships between variables and failed to advance the follow-up tracking of the study participants. Second, all data were collected by self-report questionnaires, which inevitably introduces subjective bias into the results. The results may also affected by recall and reporting biases. Moreover, the MASQ is a self-report scale. Self-evaluations may reflect daily life more accurately than neuropsychological tests but can be influenced by emotion and self-esteem (54), as well as by individual biases regarding cognitive abilities or social desirability (55). Consequently, the MASQ possesses a strong subjective component. Third, the sample size had a high proportion of females in the gender and a high proportion of medical students, limiting the generalizability of the findings. Compared to males, the female group exhibited higher average levels of perceived stress, cognitive and emotional stimulation, along with higher rates of positive screens for anxiety disorders, comorbidity of anxiety and depression, and prevalence of psychological distress (56). So we conducted ANOVA separately for males and females, and we controlled for gender as a covariate in the GLM to reduce bias. The sample contained a relatively high proportion of medical students. Students in medical majors experience higher levels of anxiety and subclinical depressive symptoms, along with lower quality of life, and depressive and anxiety symptoms are associated with poor sleep quality (57). Therefore, this study also controlled for academic major as a covariate to reduce potential bias. More in-depth longitudinal and intervention studies are needed in the future to further confirm the reliability of this finding.

## 5 Conclusion

Overall, our study reveals the interconnected roles of sleep duration, sleep disturbances, and cognitive functioning among college students. We establish that sleep duration is strongly associated with sleep disturbances, and that these sleep disturbances have distinct associations with different cognitive functions. This underscores the crucial need for more detailed exploration to direct future intervention strategies. Future studies should adopt a longitudinal approach in order to explore the causal relationship between sleep and cognition and delve deeper into the biological mechanisms underlying the link between sleep and cognitive functioning in order to provide a broader range of sleep strategies for the public.

## Data Availability

The original contributions presented in the study are included in the article/**Supplementary Material**. Further inquiries can be directed to the corresponding author.
